# Tooth Loss and Cardiovascular Disease Mortality Risk – Results from the Scottish Health Survey

**DOI:** 10.1371/journal.pone.0030797

**Published:** 2012-02-20

**Authors:** Richard G. Watt, Georgios Tsakos, Cesar de Oliveira, Mark Hamer

**Affiliations:** Department of Epidemiology and Public Health, University College London, London, United Kingdom; Innsbruck Medical University, Austria

## Abstract

**Background:**

Tooth loss is associated with increased cardiovascular disease (CVD) mortality risk. This association may however be due to residual confounding. We aimed to assess whether tooth loss is associated with specific CVD mortality endpoints in a national population sample adjusting for potential confounders.

**Methods and Results:**

We used a prospective cohort design and data from the Scottish Health Survey. We combined data from surveys in 1995, 1998, 2003 and linked this to mortality records. Dental status was classified through self-reports as natural teeth only, natural teeth and dentures, and no natural teeth (edentate). Cox proportional hazards models were used to estimate risk of CVD mortality by dental status adjusting for potential confounders. The sample consisted of 12871 participants. They were followed for 8.0 (SD: 3.3) years. During 103173 person-years, there were 1480 cases of all-cause mortality, 498 of CVD, and 515 of cancer. After adjusting for demographic, socio-economic, behavioural and health status, edentate subjects had significantly higher risk of all-cause (HR, 1.30; 95% CI, 1.12,1.50) and CVD mortality (HR, 1.49; 95% CI, 1.16,1.92) compared to subjects with natural teeth only. Dental status was not significantly associated with cancer mortality in fully adjusted analysis. Further analysis for CVD mortality showed that in the fully adjusted model, edentate subjects had 2.97 (95% CI, 1.46, 6.05) times higher risk for stroke-related mortality.

**Conclusions:**

In a national population sample of Scottish adults, being edentate was an independent predictor of total CVD mortality, although this was mainly driven by fatal stroke events.

## Introduction

Oral diseases, in particular dental caries and periodontal diseases are highly prevalent chronic conditions which have a significant impact on quality of life and require costly lifelong treatment [1], [2]. Over one hundred years ago the theory of focal sepsis, although lacking in empirical scientific evidence, hypothesised that chronic infections in the mouth caused systemic diseases [3]. In the last 20 years researchers have explored the potential association between oral diseases, and morbidity and mortality. Most of the research has investigated the association between periodontal disease and an increased risk of cardiovascular disease (CVD) and CVD mortality [4], [5], [6]. The proposed biological pathway linking periodontal diseases and CVD is thought to be through a low grade inflammatory response caused by oral bacterial infections [7], [8]. Elevated levels of inflammatory biomarkers have been implicated in atherothrombogenesis [9], [10].

Research in a variety of diverse populations has also indicated an association between tooth loss and, morbidity and mortality [9], [10], [11], [12], [13], [14], [15], [16]. Population cohort studies with follow-up periods varying from 5–57 years have shown an association between tooth loss and all-cause mortality [11], [12], [13], [14], and CVD mortality [11], [13], [14], [15]. More limited evidence exists in relation to tooth loss and the subsequent development of stroke [11], [16], [17], and certain cancers [11], [18]. A recent Swedish cohort study found a dose dependent linear relationship between number of teeth, but not clinical measures of periodontal disease, and both all-cause and CVD mortality at 12 years follow-up [14]. The suggested potential pathways between tooth loss and general health is either through impaired masticatory function leading to poor nutritional status [19], [20], [21], a recognised risk factor for CVD [22], or through inflammatory responses associated with past periodontal diseases [23]. The main cause of tooth loss in middle aged and older adults is periodontal diseases [24]. However although there is increasing epidemiological evidence linking poor oral health in the development of chronic diseases and premature mortality, it has been argued that these associations are merely due to residual confounding due to imprecise measurement of important risk factors of systemic disease such as socioeconomic background and smoking [19], [25]. Indeed many of the previous studies exploring the link between tooth loss and systemic disease have been conducted in selected samples and have failed to control adequately for socioeconomic, behavioural and general health status.

Scotland suffers from very high levels of CVD, and has one of the highest rates of premature death in Western Europe [26]. The oral health of the Scottish population is also very poor with higher levels of total tooth loss (edentate) amongst adults compared to other similar countries [27]. The Scottish population therefore provides a suitable sample to investigate the potential link between tooth loss and mortality. Using data from a national sample of Scottish adults, the aim of this study was therefore to assess the association between tooth loss and mortality rates, with a particular focus on specific CVD mortality endpoints including stroke and coronary heart disease (CHD). In addition, we examined the potential mediating effects of inflammation, indicated by levels of C-reactive protein (CRP) and nutritional status measured as body mass index (BMI) on this association, in a subset of participants.

## Methods

### Study design and participants

The Scottish Health Survey is a cross-sectional survey that is typically conducted serially every 3–5 years, that draws a nationally representative sample of the general population living in Scottish households. For the present analysis we combined data from separate surveys sampled in 1995, 1998 and 2003 (comprising 28.6%, 36.3%, 35.1% of the present sample, respectively) in adults aged 35 years and older as previously described [28]. We linked these data to mortality records; thus the analyses were based on a prospective cohort design. Participants from the different survey years contributed data only once and were comparable in terms of demographics and risk factors.

### Ethics statement

Participants gave informed written consent to take part in the study and for their information to be stored and used for research. Ethical approval was obtained from the London Research Ethics Council. Detailed information on the survey methods can be found elsewhere [29].

### Baseline assessment

Survey interviewers visited eligible households and collected data on demographic and health behaviour variables. Height and weight were measured according to standardised protocols for the calculation of body mass index (weight [kg]/height [m^2^]). Height was measured using a portable stadiometer with a sliding head plate and weight using Soehnle and Tanita electronic scales with a digital display. On a separate visit, nurses collected blood in consenting adults, and information on medical and dental histories. Dental status was classified through self-reports as natural teeth only, natural teeth and dentures, and no natural teeth (edentate).

### Mortality follow-up

The surveys were linked to a patient-based database containing information on mortality with follow-up until December 2007 (Information Services Division, Scotland). Classification of the underlying cause of death was based on information collected on the death certificate together with any additional information provided subsequently by the certifying doctor. We examined cause specific mortality according to International Classification of Diseases - Version 9 (ICD-9) and 10 (ICD-10); including cardiovascular causes (ICD-9: 390–459; ICD-10: I01-I99) and cancer (ICD-9: 140–239; ICD-10: C00-D48). The linkage was successful in 79.7% of the sample and the remainder did not consent or were lost to follow-up.

### Inflammatory biomarkers

Peripheral blood was collected in serum tubes and spun at room temperature. All blood samples were frozen at −70°C until assay. The analysis of CRP levels from serum was performed using the N Latex high sensitivity CRP mono immunoassay on the Behring Nephelometer II analyser. The limit of detection was 0.17 mg/L and the coefficient of variation (CV) was less than 6% for this assay. The analyses were carried out according to Standard Operating Procedures by State Registered Medical Laboratory Scientific Officers.

### Statistical analysis

Cox proportional hazards models were used with months as the time scale to estimate the risk of mortality according to dental status (categorized as; only natural teeth [referent], natural teeth and denture(s), edentate). Study members that survived were censored at 31^st^ December 2007. In preliminary analyses, there were no clear differences in our results between men and women, so the data were pooled, and sex and age-adjusted in the basic model. In further models we adjusted for potential confounders including socioeconomic group (using the Registrar General Classification; I/II professional/intermediate, III skilled non-manual/skilled manual, IV/V part-skilled/unskilled) and marital status (yes/no). We then further added health behaviours: frequency of weekly physical activity (split into tertiles), smoking (never, previous, current), weekly alcohol intake (never, ex-drinker, trivial; moderate 1–21 units; heavy ≥21 units), and body mass index category (>25, 25–30, >30 kg/m^2^). Lastly, we adjusted for health status including self-rated health (very good, good, fair, bad, very bad), doctor diagnosed diabetes and hypertension from doctor's diagnosis or clinic blood pressure readings. In a separate analysis we examined the potential mediating effects of inflammation (CRP) and nutritional status (using body mass index as an indicator). In these models we added CRP or BMI into a basic model (adjusted for age, sex, smoking, socioeconomic group) and estimated the proportion of risk explained as follows: (HR_basic model_−HR_adjusted_)/HR_basic model_−1)×100. The proportional hazards assumption was examined in two ways; first by comparing the survival curves for each level of exposure over the duration of follow-up, and second by determining whether the interaction term exposure x log (follow-up period) was non-significant. We used ANOVA tests to examine continuous variables and chi-square tests to examine univariable relationships of the covariates with the exposure measure. All analyses were performed using SPSS (version 14) and all tests of statistical significance were based on two-sided probability (p<0.05).

## Results

The original sample consisted of 16144 participants, although after the exclusion of non-respondents (n = 280), and participants with no linkage data (n = 2993), the final analytical sample consisted of 12871 individuals. Participants excluded because of missing demographics did not differ significantly on key characteristics such as age (51.9 vs 53.1 yrs, p = 0.09). However, those excluded because of missing linkage were younger (48.8 vs 54.1 yrs, p<0.001) and less likely to report edentate status (12.2 vs. 25.3%, p<0.001) compared with the main sample. Because a substantial proportion of the sample (n = 1457) had missing data on BMI, the missing cases were recoded with a dummy variable in order to retain them in the analyses. At baseline, the sample consisted of 6016 participants with natural teeth only, 3615 with both natural teeth and dentures, and 3240 edentate participants. Baseline characteristics of the study population according to dental status are given in Table 1. Poorer dental status was associated with older individuals, women, lower socioeconomic group, not being married, smoking, physical inactivity, less alcohol intake, obesity, poor self-rated health, diabetes, hypertension and high CRP (p<0.001 in all cases). The sample was followed up for a mean period of 8.0 (sd: 3.3) years. During 103173 person-years of follow-up, 1480 cases of all-cause mortality were recorded; an incidence rate of 14.3/1000 persons per year. In relation to specific causes of mortality, 498 people died from CVD (incidence rate: 4.8/1000) and 515 from cancer (incidence rate: 5.0/1000).

**Table 1:** Characteristics of the study population in relation to dental status (n = 12871).

Variable	Dental status (n (%))			
	Only natural teeth (n = 6016)	Natural/dentures (n = 3615)	Edentate (n = 3240)	*P*-trend
Mean Age (SD)	48.7±10.6	55.3±11.7	63.0±10.8	<.001
Men	2830 (47.0)	1636 (45.3)	1236 (38.1)	<.001
Upper SEG	2137 (35.5)	955 (26.4)	550 (17.0)	<.001
Married	3969 (66.0)	2272 (62.8)	1696 (52.3)	<.001
Smokers	1578 (26.2)	1201 (33.2)	1280 (39.5)	<.001
Physical inactivity	2033 (33.8)	1502 (41.5)	1797 (55.5)	<.001
Moderate alcohol	3910 (65.0)	2056 (56.8)	1500 (46.3)	<.001
Obesity	1297 (21.6)	831 (23.0)	860 (26.5)	<.001
Poor self rated health	386 (6.4)	348 (9.6)	572 (17.6)	<.001
Diabetes	174 (2.9)	147 (4.1)	253 (7.8)	<.001
Hypertension	1353 (22.0)	1123 (31.1)	1265 (39.0)	<.001
†High CRP (≥3 mg/L)	792 (27.8)	612 (37.0)	757 (50.4)	<.001

Upper socio-economic group (SEG) refers to participants in professional/intermediate band of the Registrar General Classification; Physical inactivity refers to participants in the lowest tertile; Obesity defined as body mass index ≥30 kg/m^2^; Poor self rated health refers to participants giving rating of bad/very bad; moderate alcohol defined as consuming >1<21 units per week.

† C-reactive protein (CRP) measured in a sub-sample of participants (n = 6005).

**Figure 1:**
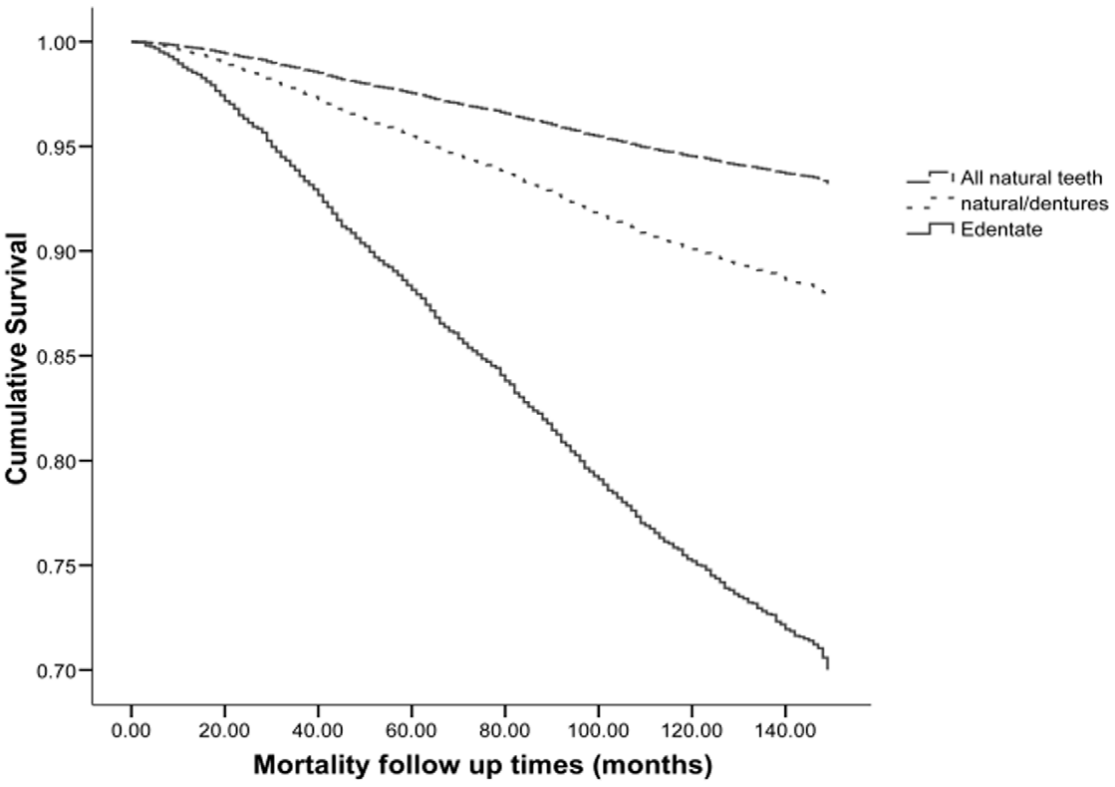
Cumulative survival plot of dental status and risk of all cause mortality.

The cumulative survival plot for all-cause mortality shows higher mortality for poorer dental status, particularly for edentate individuals (Figure 1). In age and sex adjusted analyses, being edentate was a significant predictor for mortality risk. Compared to participants with natural teeth only, edentate individuals had significantly higher hazard ratios for all-cause, CVD, and cancer mortality (Table 2, model 1). These estimates became gradually lower after sequential adjustment for socioeconomic group and marital status (Table 2, model 2), health behaviours (Table 2, model 3), and self-rated health and diagnosed diabetes (Table 2, model 4). For all three causes of mortality, the largest attenuation was observed when health behaviours were entered in the model. Demographic, socio-economic, behavioural and health status confounders collectively explained 69% of the increased risk among edentate subjects for all-cause mortality and 60% for the respective increased risk for CVD mortality. Even after adjusting for all these potential confounders, edentate participants at baseline had significantly higher risk for all-cause mortality (HR: 1.30; 95% CI 1.12, 1.50) and CVD mortality (HR: 1.49; 95% CI 1.16, 1.92), compared to participants with natural teeth only. Dental status was not associated with significantly increased risk for cancer mortality in the model adjusting for health behaviours and the fully adjusted one (HR: 1.11; 95% CI 0.88, 1.40). When we repeated these analyses removing participants with missing BMI data the results were unchanged; edentate participants at baseline had significantly higher risk for all-cause mortality (HR: 1.30; 95% CI 1.11, 1.52) and CVD mortality (HR: 1.49; 95% CI 1.13, 1.95) compared to those with natural teeth only.

**Table 2:** Hazard ratios (95% CI) for the relation between dental status and risk of death (n = 12,871).

	Event/N	Model 1 HR (95% CI)	Model 2 HR (95% CI)	Model 3 HR (95% CI)	Model 4 HR (95% CI)
**All cause mortality**					
Only natural teeth	356/6016	1.00	1.00	1.00	1.00
Natural/dentures	366/3615	1.12 (0.97–1.30)	1.11 (0.95–1.28)	1.01 (0.87–1.17)	0.98 (0.85–1.14)
Edentate	758/3240	1.90 (1.65–2.18)	1.74 (1.51–2.00)	1.40 (1.22–1.62)	1.30 (1.12–1.50)
**Cardiovascular disease death**					
Only natural teeth	103/6016	1.00	1.00	1.00	1.00
Natural/dentures	116/3615	1.17 (0.89–1.53)	1.14 (0.87–1.49)	1.08 (0.82–1.41)	1.02 (0.78–1.34)
Edentate	279/3240	2.21 (1.73–2.82)	2.00 (1.55–2.56)	1.63 (1.27–2.11)	1.49 (1.16–1.92)
**Cancer death**					
Only natural teeth	147/6016	1.00	1.00	1.00	1.00
Natural/dentures	124/3615	0.94(0.73–1.19)	0.92 (0.72–1.18)	0.83 (0.65–1.07)	0.82 (0.64–1.05)
Edentate	244/3240	1.51 (1.21–1.89)	1.45 (1.15–1.83)	1.17 (0.92–1.48)	1.11 (0.88–1.40)

Mean follow up = 8.0±3.3 yrs.

Model 1: adjusted for age and sex.

Model 2: additional adjustment for socioeconomic group (professional/intermediate; skilled non-manual; skilled manual; part-skilled/unskilled; other), marital status (yes/no).

Model 3: additional adjustment for physical activity (tertile of low; medium; high), smoking (never; previous; current), alcohol (never, ex-drinker, trivial; moderate <21 units; heavy ≥21 units/wk), body mass index category (<25; 25–30; ≥30 kg/m^2^).

Model 4: additional adjustment for self rated health (very good; good; fair; bad; very bad), physician diagnosed diabetes (yes; no), hypertension (physician diagnosed or clinic BP>140/90 mmHg).

In further analysis we explored in particular the association between dental status and CVD mortality, by partitioning the latter into two main causes: CHD and stroke. Being edentate was a significant predictor of both CHD and stroke mortality in models sequentially adjusting for age-sex, socioeconomic position, and health behaviours, but the hazard ratios were much higher for mortality due to stroke (Table 3). In the fully adjusted model, additionally controlling for general health status, there was a gradient in mortality risk from stroke for each group with poorer dental status. Compared to participants with natural teeth only at baseline, those who had both natural teeth and dentures were at 2.09 (95% CI 1.01, 4.34) times higher risk and the edentate had 2.97 (95% CI 1.46, 6.05) times higher risk for stroke-related mortality at follow-up. On the contrary, the respective association between dental status and CHD was not significant in the fully adjusted model (Table 3).

**Table 3:** Hazard ratios (95% CI) for the relation between dental status and risk of CVD specific death (n = 12,871).

	Event/N	Model 1 HR (95% CI)	Model 2 HR (95% CI)	Model 3 HR (95% CI)	Model 4 HR (95% CI)
**Coronary heart disease**					
Only natural teeth	74/6016	1.00	1.00	1.00	1.00
Natural/dentures	71/3615	1.00 (0.72–1.40)	0.99 (0.71–1.39)	0.92 (0.66–1.29)	0.87 (0.62–1.22)
Edentate	152/3240	1.75 (1.29–2.36)	1.63 (1.19–2.22)	1.35 (0.99–1.86)	1.22 (0.89–1.68)
**Stroke**					
Only natural teeth	12/6016	1.00	1.00	1.00	1.00
Natural/dentures	24/3615	2.44 (1.18–5.03)	2.32 (1.12–4.79)	2.19 (1.06–4.54)	2.09 (1.01–4.34)
Edentate	56/3240	4.61 (2.32–9.16)	3.82 (1.90–7.68)	3.23 (1.59–6.58)	2.97 (1.46–6.05)

Model 1: adjusted for age and sex.

Model 2: additional adjustment for socioeconomic group (professional/intermediate; skilled non-manual; skilled manual; part-skilled/unskilled; other), marital status (yes/no).

Model 3: additional adjustment for physical activity (tertile of low; medium; high), smoking (never; previous; current), alcohol (never, ex-drinker, trivial; moderate <21 units; heavy ≥21 units/wk), body mass index category (<25; 25–30; ≥30 kg/m^2^).

Model 4: additional adjustment for self rated health (very good; good; fair; bad; very bad), physician diagnosed diabetes (yes; no), hypertension (physician diagnosed or clinic BP>140/90 mmHg).

As previous studies have suggested effect modification by age, sex and smoking status in relation to dental status and mortality [23], we performed various sensitivity analyses. The findings of the sensitivity analysis demonstrated the robustness of the association between dental status and all-cause mortality risk, as the results were consistent across age groups, for both sexes, and for the different smoking categorisations (non-smokers, past smokers, current smokers). Furthermore, the association was also confirmed even when removing all deaths that had occurred in the first 2 years of the follow-up period (Table S1). In addition, when we removed 725 participants with clinically confirmed CVD at baseline (assessed from retrospective hospital records), the results were marginally changed and if anything strengthened; edentate participants at baseline had significantly higher risk for all-cause mortality (HR: 1.37; 95% CI 1.18, 1.60) and CVD mortality (HR: 1.69; 95% CI 1.27, 2.27) compared to those with natural teeth in fully adjusted models (Table S2).

Finally, to assess the potential mediating effects of inflammatory biomarkers and nutritional status on the association between dental status and mortality risk, we re-ran the survival analysis in a sub-sample of participants with available biological and anthropometric data. A biological marker of inflammation, CRP, and an indicator of nutritional status, BMI, did not have any effect on the association between dental status and all-cause and CVD mortality, though these analyses were carried out in less than half the original analytical sample (n = 6005) due to non-availability of data (Table 4).

**Table 4:** The association between dental status, biological mediators, and mortality risk. (n = 6005).

	Event/N	Model 1 HR (95% CI)	Model 2 HR (95% CI)	Model 3 HR (95% CI)
**All cause death**				
Only natural teeth	129/2851	1.00	1.00	1.00
Natural/dentures	147/1653	1.07 (0.84–1.37)	1.06 (0.83–1.35)	1.08 (0.85–1.38)
Edentate	336/1501	1.63 (1.29–2.04)	1.58 (1.25–1.98)	1.65 (1.31–2.07)
**CVD death**				
Only natural teeth	43/2851	1.00	1.00	1.00
Natural/dentures	44/1653	0.94 (0.61–1.44)	0.91 (0.59–1.40)	0.95 (0.61–1.46)
Edentate	123/1501	1.75 (1.19–2.59)	1.69 (1.15–2.49)	1.76 (1.19–2.59)

Model 1: adjusted for age, sex, socioeconomic group, smoking.

Model 2: adjusted for age, sex, socioeconomic group, smoking+log C-Reactive Protein.

Model 3: adjusted for age, sex, socioeconomic group, smoking+body mass index category (<25; 25–30; ≥30 kg/m^2^).

## Discussion

In this prospective cohort study of a national sample of Scottish adults we have shown that poorer dental status, edentulousness in particular, is an independent predictor of both all-cause and CVD mortality. Even after extensive adjustment for recognised confounders, edentate participants had a significantly elevated risk of all-cause and CVD mortality, but not cancer mortality. Interestingly when we further assessed the association between dental status and CVD mortality, it was stroke-related, rather than CHD-related, deaths that were affected by dental status. In the fully adjusted models, edentate individuals had almost a three fold elevated risk of stroke mortality than participants who had some natural teeth. The robustness of the association between poor dental status and all-cause mortality was demonstrated in sensitivity analysis with consistent results across age groups, between sexes and by smoking status. However our analysis provided very limited evidence of a mediating role for either inflammatory or nutritional factors on this association.

Our findings confirm previous studies which have shown a small but significant association between tooth loss and all-cause and CVD deaths after controlling for a range of potential confounding factors [11], [12], [13], [14], [15]. Indeed the elevated risk estimates we have reported for CVD mortality (HR 1.50; 95% CI 1.16,1.93) are very similar to the summary estimates (RR 1.34; 95% CI 1.10, 1.63) from a recent meta-analysis assessing the association between CHD/CVD events and tooth loss [5]. Unlike large cohort studies conducted in China and the US [11], [18], we did not find a significant association between tooth loss and cancer risk in this Scottish sample. This is most likely due to the smaller sample size in our study. However our more striking finding in relation to fatal strokes are in line with previous population cohort studies. Wu and colleagues showed that periodontal disease, including tooth loss, was positively associated to fatal stroke (HR 2.90; 95% CI 1.49,5.62) in the longitudinal element of the first US National Health and Nutrition Examination Survey [30]. In a more recent Danish study, edentulous subjects had a significantly increased risk of fatal and non-fatal strokes (HR 3.25; 95% CI 1.48,7.14) than those with remaining natural teeth [16]. Data from the Glasgow Alumni cohort showed that subjects with nine or more missing teeth at baseline had an increased risk of stroke mortality (HR 2.79; 95% CI 1.30,5.97) at follow-up [15].

Previous studies and reviews have discussed potential causal mechanisms that might explain the association between tooth loss and mortality. People with fewer teeth and especially those who are edentate may have impaired masticatory function which limits their dietary choices and affects their nutritional status [19], [20], [21]. In Scotland due to very high levels of dental caries, the complete removal of all natural teeth was a relatively common treatment for young adults. Therefore in this sample many of the older subjects may have been edentate for many years leading to long-term nutritional disadvantage. Poor nutritional status is an established risk factor for CVD [22]. An alternative inflammatory pathway has been proposed which includes the direct effects of oral infective agents collaborating in atheroma formation, indirect effects of chronic inflammation and common genetic predisposition to periodontal disease and vascular diseases [31], [32], [33], [34]. In our analysis we attempted to explore the potential mediating role of both nutritional and inflammatory factors on the association between dental status and both all-cause, and CVD mortality, but found no evidence of these roles. Nevertheless, it should be noted that we have only tested the inflammatory hypothesis using one biomarker of systemic inflammation, and the inclusion of further detailed measures of the immune system, such as inflammatory cytokines (eg, interleukin-6, tumor necrosis factor alpha) might have provided a more detailed analysis. It is difficult to explain the stronger association we have found for stroke than CHD mortality. Previous research which found a similar finding suggested that the anatomical relationship between the maxilla and the cavernous sinus or vascular complex or the common lymphatic drainage could facilitate local spreading of infectious agents [16]. In addition, whilst coronary heart disease and stroke share some common risk factors such as male sex, cholesterol, and smoking, there are also unique predictors for each endpoint [35], that might reflect subtle differences in the pathogenesis of these diseases [36]. An alternative, but largely under researched common pathway potentially linking dental status and mortality are shared psycho-social determinants for both oral and general chronic conditions.

Although this study was conducted in a national population sample and had a robust methodology in terms of good quality follow-up data and thorough analysis which controlled for potential confounders, our results need to be interpreted in the context of several potential limitations. Although the Scottish Health Survey is representative of the general population, the data presented in this study may not be, due to missing data and loss of information on data linkage. The individual's lost to follow-up were younger and less likely to report being edentate than the main sample. Our self-reported measures of dental status were rather crude indicators of dental disease. Clinical data on the actual number of natural teeth as well as the condition of the teeth and periodontal tissues would have provided more detailed information and a more accurate measure of dental status. We also only had information on the dental status of the sample at baseline. Over the 8-year follow-up period the dental status of some of the sample would have deteriorated as a small number would have become edentulous over this time frame. This would have potentially diluted the effects, thus results presented herein might reflect a conservative estimate of the true association. It would have been interesting to separate ischemic and hemorrhagic strokes in our analysis but this was not possible as 46 out of the 92 stroke events had a non-specified stroke ICD code, highlighting also the limitation of the ICD coding system. Finally, due to a significant reduction in the sample due to non-availability of relevant data, our analysis on the potential mediating role of inflammatory and nutritional factors may lack necessary power. The study would also have benefited from availability of a more complete array of inflammatory and nutritional biomarkers.

In conclusion, poor dental status, in particular being edentate was an independent predictor of higher all-cause, CVD and stroke mortality even after adjustment for demographic, socio-economic, behavioural and health status in this national sample of Scottish adults. Well designed experimental population trials are needed to further investigate this association and to determine if dental preventive and treatment interventions have a beneficial effect on mortality risk.

## Supporting Information

Table S1 **Sensitivity analyses based on all cause mortality.**
(DOCX)

Table S2 **Hazard ratios (95% CI) for the relation between dental status and risk of death excluding participants with existing CVD (n = 12,146).**
(DOCX)
