# Supplementary material for: Tooth Loss and Cardiovascular Disease Mortality Risk – Results from the Scottish Health Survey
Source: PLoS One. 2012 Feb 20;7(2):e30797. doi: 10.1371/journal.pone.0030797 (PMC3282705; doi:10.1371/journal.pone.0030797)
Supplement: Table S1 — Sensitivity analyses based on all cause mortality. (DOCX) [file pone.0030797.s001.docx]

Table S1. Sensitivity analyses based on all cause mortality

1. Effect modification by age, sex, smoking status.

|  | Events/N | Age & sex adjusted |
| --- | --- | --- |
|  |  | HR (95% CI) |
| **Stratified by Age** |  |  |
| *<65 yrs old* |  |  |
| Only natural teeth | 281/ 5497 | 1.00 |
| Natural teeth/dentures | 229/ 2823 | 1.11 (0.93 – 1.33) |
| Edentate | 326/ 1824 | 1.78 (1.50 – 2.11) |
|  |  |  |
| *≥65yrs old* |  |  |
| Only natural teeth | 75/ 519 | 1.00 |
| Natural teeth/dentures | 137/ 792 | 1.16 (0.87 – 1.53) |
| Edentate | 432/ 1416 | 2.07 (1.62 – 2.66) |
|  |  |  |
| **Stratified by sex** |  |  |
|  |  |  |
| *Men* |  |  |
| Only natural teeth | 232/ 2830 | 1.00 |
| Natural teeth/dentures | 205/ 1636 | 1.06 (0.88 – 1.28) |
| Edentate | 349/ 1236 | 1.75 (1.46 – 2.09) |
|  |  |  |
| *Women* |  |  |
| Only natural teeth | 124/ 3186 | 1.00 |
| Natural teeth/dentures | 161 /1979 | 1.24 (0.98 – 1.57) |
| Edentate | 409/ 2004 | 2.13 (1.72 – 2.65) |
|  |  |  |
| **Stratified by smoking** |  |  |
|  |  |  |
| *Non-smokers* |  |  |
| Only natural teeth | 107/2835 | 1.00 |
| Natural teeth/dentures | 100/1307 | 1.16 (0.87-1.53) |
| Edentate | 126/847 | 1.52 (1.14-2.03) |
|  |  |  |
| *Ex-smokers* |  |  |
| Only natural teeth | 111/1603 | 1.00 |
| Natural teeth/dentures | 124/1107 | 1.03 (0.79-1.34) |
| Edentate | 261/1113 | 1.62 (1.27-2.05) |
|  |  |  |
| *Smokers* |  |  |
| Only natural teeth | 139/1578 | 1.00 |
| Natural teeth/dentures | 143/1201 | 0.90 (0.71-1.14) |
| Edentate | 371/1280 | 1.34 (1.08-1.66) |

1. Removing all deaths in first 2 yrs of follow up (n=12,620).

|  | Events/N | Age & sex adjusted |
| --- | --- | --- |
|  |  | HR (95% CI) |
|  |  |  |
| Only natural teeth | 327/5961 | 1.00 |
| Natural teeth/dentures | 328/3555 | 1.12 (0.95-1.31) |
| Edentate | 665/3104 | 1.88 (1.62-2.19) |
