# Supplementary material for: Tooth Loss and Cardiovascular Disease Mortality Risk – Results from the Scottish Health Survey
Source: PLoS One. 2012 Feb 20;7(2):e30797. doi: 10.1371/journal.pone.0030797 (PMC3282705; doi:10.1371/journal.pone.0030797)
Supplement: Table S2 — Hazard ratios (95% CI) for the relation between dental status and risk of death excluding participants with existing CVD (n = 12,146). (DOCX) [file pone.0030797.s002.docx]

Table S2. Hazard ratios (95% CI) for the relation between dental status and risk of death excluding participants with existing CVD (n=12,146).

|  | **Event/N** | **Model 1 HR (95% CI)** | **Model 2 HR (95% CI)** | **Model 3 HR (95% CI)** | **Model 4 HR (95% CI)** |
| --- | --- | --- | --- | --- | --- |
| **All cause mortality** |  |  |  |  |  |
| Only natural teeth | 313/5831 | 1.00 | 1.00 | 1.00 | 1.00 |
| Natural /dentures | 319/3417 | 1.14 (0.97-1.34) | 1.12 (0.95-1.30) | 1.30 (0.81-1.21) | 0.99 (0.85-1.17) |
| Edentate | 652/2898 | 2.01 (1.73-2.33) | 1.82 (1.56-2.11) | 1.48 (1.27-1.73) | 1.37 (1.18-1.60) |
|  |  |  |  |  |  |
| **Cardiovascular disease death** | |  |  |  |  |
| Only natural teeth | 77/5831 | 1.00 | 1.00 | 1.00 | 1.00 |
| Natural/dentures | 92/3417 | 1.27 (0.93-1.73) | 1.23 (0.90-1.67) | 1.16 (0.85-1.57) | 1.11 (0.81-1.51) |
| Edentate | 224/2898 | 2.55 (1.93-3.37) | 2.21 (1.66-2.94) | 1.84 (1.37-2.47) | 1.69 (1.27-2.27) |
|  |  |  |  |  |  |
| **Cancer death** |  |  |  |  |  |
| Only natural teeth | 138/5831 | 1.00 | 1.00 | 1.00 | 1.00 |
| Natural/dentures | 117/3417 | 0.94 (0.73-1.19) | 0.92 (0.72-1.18) | 0.83 (0.65-1.07) | 0.82 (0.64-1.05) |
| Edentate | 217/2898 | 1.54 (1.21-1.89) | 1.45 (1.15-1.83) | 1.17 (0.92-1.48) | 1.14 (0.89-1.46) |

Model 1: adjusted for age and sex

Model 2: additional adjustment for socioeconomic group (professional/intermediate; skilled non-manual; skilled manual; part-skilled/unskilled; other), marital

status (yes/no)

Model 3: additional adjustment for physical activity (tertile of low; medium; high), smoking (never; previous; current), alcohol (never, ex-drinker, trivial; moderate <21 units; heavy ≥21 units/wk), body mass index category (<25; 25-30; ≥30 kg/m^2^ )

Model 4: additional adjustment for self rated health (very good; good; fair; bad; very bad), physician diagnosed diabetes (yes; no), hypertension (physician diagnosed and/or clinic BP >140/90 mmHg)
